# Cryopreservation of reproductive material before cancer treatment: a qualitative study of health care professionals’ views about ways to enhance clinical care

**DOI:** 10.1186/s12913-017-2292-2

**Published:** 2017-05-10

**Authors:** Karin Hammarberg, Maggie Kirkman, Catharyn Stern, Robert I. McLachlan, Debra Gook, Luk Rombauts, Beverley Vollenhoven, Jane R. W. Fisher

**Affiliations:** 1 Jean Hailes Research Unit, School of Public Health and Preventive Medicine, Monash University, 549 St Kilda Rd, Melbourne, Victoria 3004 Australia; 2 Melbourne IVF, Melbourne, Victoria 3002 Australia; 3 Reproductive Services, Royal Women’s Hospital, Parkville, Victoria 3052 Australia; 4 Department of Obstetrics and Gynaecology, University of Melbourne, Melbourne, Victoria 3010 Australia; 5 Monash IVF, Clayton, Victoria 3163 Australia; 6 Andrology Australia, Melbourne, Victoria 3163 Australia; 7 Hudson Institute of Medical Research, Clayton, Victoria 3163 Australia; 8 Monash Health, Clayton, Victoria 3163 Australia; 9 Department of Obstetrics and Gynaecology, Monash University, Clayton, Victoria 3163 Australia

**Keywords:** Cancer, Fertility preservation, Cryopreservation, Service provision

## Abstract

**Background:**

Cancer treatment can diminish fertility in women and men. The need for fertility preservation is growing as increasing numbers of people survive cancer. Cryostorage of reproductive material to preserve potential for conception for cancer survivors has moved from being experimental to being a part of clinical management of women and men who are diagnosed with cancer in their reproductive years. There is little existing evidence about how fertility preservation services can be enhanced to meet the complex needs of patients who are diagnosed with cancer in their reproductive years. The aim of this research was to inform clinical practice development by drawing on the collective experience and knowledge of staff at well-established clinics that offer fertility preservation before cancer treatment.

**Methods:**

A qualitative research model was adopted using semi-structured interviews with professionals involved in the care of people who freeze reproductive material before cancer treatment. In the state of Victoria, Australia, two large assisted reproductive technology (ART) centres have been providing fertility preservation services for more than two decades. An invitation to participate in a semi-structured interview about clinical care in the context of fertility preservation was emailed to past and current staff members. To capture diverse perspectives, informants were sought from all relevant professions: fertility specialists, andrologists, nurses, embryologists/scientists, counsellors, and administrative staff. Transcripts were analysed thematically.

**Results:**

Thirteen key informants were interviewed from August 2013 to February 2014. The identified themes relating to enhancing clinical care in a fertility preservation service were *communication between oncology and ART specialists*; *managing urgency*; *managing patients’ expectations*; *establishing and implementing protocols, systems, and data bases*; and *maintaining contact with patients*.

**Conclusion:**

The collective knowledge of this study’s informants, who represent multidisciplinary teams with more than two decades’ experience in fertility preservation, yields important insights into strategies that fertility preservation services can employ to promote the integration of oncology and fertility care, the psychosocial care of patients, data recording and monitoring, and reporting of outcomes.

**Electronic supplementary material:**

The online version of this article (doi:10.1186/s12913-017-2292-2) contains supplementary material, which is available to authorized users.

## Background

Many people of reproductive age who are diagnosed with cancer hope to have children after completing treatment [1, 2]. Cancer treatment can diminish fertility in women and men [3, 4]. Fertility preservation techniques have been developed in response to this, and an increasing number of assisted reproductive technology (ART) clinics offer this service to people requiring treatment for cancer.

Most techniques for retaining potential for parenthood after cancer involve cryopreservation of reproductive material. The disease, the urgency of treatment, and patients’ characteristics (e.g., age and relationship status) can limit the options [5]. Sperm banking is a well-established method to preserve fertility in men [6]. For some types of cancer, the availability of frozen sperm doubles the chance of fatherhood after cancer treatment [4]. The science of preserving female fertility is more recent and still emerging. Women can be offered cryopreservation of embryos, oocytes, or ovarian tissue. However, this is much less common than sperm banking, in part because of the greater complexity of harvesting oocytes and ovarian tissue. Harvesting oocytes for freezing, whether unfertilised or as embryos, requires hormone stimulation and the time required to stimulate the ovaries to produce multiple oocytes delays initiation of cancer treatment [7, 8].

The likelihood of pregnancy resulting from cryopreserved oocytes has improved as a result of the increasing use of the vitrification freezing technique, with pregnancy rates now similar to those achieved with fresh oocytes [9]. Whether this can be extrapolated to cancer patients is unknown; few pregnancies have been reported in women who conceived with oocytes that were frozen before they had cancer treatment [10, 11]. Ovarian tissue freezing and transplantation are still experimental processes but practitioners contend that they are safe and appropriate for girls and women diagnosed with cancer for whom oocyte harvesting may not be possible or desirable [12–15] and births using this technique have been reported [16]. New techniques are also being developed for men and boys, including testicular tissue cryopreservation and grafting [17].

The need for fertility preservation is growing as increasing numbers of people survive cancer [18]. Furthermore, in some jurisdictions, partners of people who succumb to cancer can use the deceased partner’s gametes for posthumous conception [19]. The desire to have children and the potential loss of fertility as a result of cancer treatment can cause significant distress for young people, particularly women, who are diagnosed with cancer [20]. Many cancer survivors will not have begun to fulfil or have not achieved their reproductive aspirations. Pregnancy and parenthood can represent normality, a focus on the positive, happiness, and fulfilment [1, 21]; people who lose their fertility as a result of cancer and its treatments can feel a sense of profound and preoccupying sorrow [22, 23]. Cryopreservation of reproductive material gives hope of averting reproductive loss and protecting against its accompanying grief [24]. Long-term quality of life, which encompasses fertility and parenthood, is thus of increasing importance in cancer management and the American Society for Reproductive Medicine recommends that people who are diagnosed with cancer in their reproductive years should be informed about options for fertility preservation and future reproduction [25].

While there is ample evidence about advances in the technical aspects of fertility preservation, less is known about the psychosocial aspects of fertility preservation in the context of cancer. Emerging evidence indicates that there are psychological benefits for women in being counselled about fertility preservation and the possibility of storing reproductive material [26], that counselling about fertility with the oncologist and a fertility specialist before cancer treatment reduces women’s risk of decision regret [24], and that both women and men want health professionals to enable informed decision-making by discussing the impact of gonadotoxic treatment on fertility and to refer them to specialist or therapeutic help if needed [27]. Interviews with men at risk of losing their fertility after cytotoxic treatment have found that men in this predicament appreciate the opportunity to bank their sperm but may be unprepared for the process and could need counselling [28] and that freezing sperm eases concerns about potential future fertility loss [29]. Studies assessing health professionals’ knowledge, attitudes, and practice in relation to providing fertility preservation counselling suggest that most oncologists feel ill-equipped to provide counselling because they lack knowledge about fertility preservation, appropriate fertility preservation facilities to which patients can be referred, professional links with fertility specialists, institutional guidelines and policies, and clarity about their role within the multidisciplinary team [30].

Increasingly, ART clinics offer fertility preservation as part of their service. Clinical guidelines relating to fertility preservation before cancer treatment recommend a multidisciplinary approach to information provision, counselling, and clinical care; that the potential threat to fertility posed by cancer treatment is discussed as early as possible in the treatment process; that women and men are offered fertility preservation and informed about available options; and that advice to patients takes into account the cancer diagnosis and prognosis, treatment plan, expected outcome of subsequent fertility treatment, and viability of thawed material [25, 31–33]. In addition, based on their review of the literature, Pacey and Eiser [34] make recommendations for clinical practice to preserve male fertility which include development of protocols and procedures to identify men who may benefit from fertility preservation, monitoring of uptake and outcomes of fertility preservation, and establishing systems to maintain contact with men who store sperm. To our knowledge no studies have explored how ART clinics operationalise and implement such guidelines and recommendations. The aim of this research, therefore, was to provide evidence to inform clinical practice development by drawing on the collective experience and knowledge of staff at well-established clinics offering fertility preservation for people diagnosed with cancer.

## Method

### Setting

In the state of Victoria, Australia, two large ART centres have been providing fertility preservation services for more than two decades. Between them they store reproductive material for several thousand individuals. Melbourne IVF and Monash IVF together undertake more than 90% of all fertility preservation procedures in Victoria and have significant clinical and scientific expertise in this area.

### Recruitment

Fertility preservation in the context of cancer treatment requires a multidisciplinary team and clinical practice evolves over time. In order to capture the diverse knowledge and experiences of the team, informants were sought from all relevant professions involved in establishing fertility preservation services at Melbourne IVF and Monash IVF or currently providing this service: fertility specialists, andrologists, nurses, embryologists/scientists, counsellors, and administrative staff. An invitation to participate in a semi-structured interview about the development of clinical care and their role in fertility preservation was emailed to past and current staff members who had been or currently were involved in fertility preservation. Those who expressed interest were sent a detailed explanatory statement and a consent form. An appointment was made with those who consented to be interviewed.

### Interview protocol

A discussion guide was developed, informed by the investigators’ clinical and research experience, the published literature in the field of fertility preservation care [2, 27, 35], and the aim of the study (Additional file 1). It addressed historical and current clinical care practices, forms of documentation and record keeping, and perceptions about barriers to and enablers of optimal clinical care.

### Procedure

Face-to-face interviews were conducted from August 2013 to February 2014 by KH and JF in a location chosen by the informants and audio-recorded with their consent.

### Analysis

Recordings were transcribed by a skilled transcriber and identifying details removed or amended. Transcripts were entered into NVivo 10 to assist with data management. The established techniques of thematic analysis [36] were applied in an iterative process that moved between transcripts and a developing thematic structure encompassing the development of clinical practice in counselling and information provision about fertility preservation, and the collection, storage, and later use of reproductive material in the context of cancer. Transcripts were first coded according to themes inherent in the interview guide and then examined for original themes introduced by the key informants. Each time the thematic structure was refined, all transcripts were read again to ensure that data were accurately and comprehensively represented. The thematic structure was finalised and its meaning interpreted through discussions among researchers (KH, MK, and JF). Representative quotations were then selected to illustrate the themes.

## Results

### Informant characteristics

Thirteen key informants were interviewed, of whom seven were associated with Melbourne IVF and six with Monash IVF. They represented embryologists/scientists (4), fertility specialists (3), andrologists (2), counsellors (2), and administrative staff (2). No nurses volunteered to be interviewed. To maintain confidentiality, informants are identified hereafter by their profession. Interviews lasted between 40 and 75 min.

### Themes relating to the development of clinical practice for fertility preservation

The identified themes, evident across disciplines, were *communication between oncology and ART specialists*; *managing urgency*; *managing patients’ expectations*; *establishing and implementing protocols, systems, and data bases*; and *maintaining contact with patients*. Apart from the comments about male fertility preservation offered by the two andrologists, respondents’ comments almost exclusively related to fertility preservation for women diagnosed with cancer.

### Communication between oncology and ART specialists

Informants, the fertility specialists and andrologists in particular, spoke of the fundamental need for good communication between oncologists and fertility specialists. Historically, according to informants, oncologists did not regard fertility preservation as a priority, which meant that they did not discuss fertility preservation options with their patients nor refer them to a fertility specialist. Patients who requested fertility preservation in the early days were mostly self-referred. There was consensus that referral patterns had changed over time as oncologists’ attitudes to fertility preservation moved from apprehensive to facilitative.

You know, sometimes, in the early days, a lot of these doctors weren’t comfortable about talking about fertility issues anyway, so they would shy away from bringing them up. But that’s changed, so I think that all people are pretty used to doing this now, and some of them have even made comments that—you know, actually diverting their mind onto the fertility preservation often seems to help them a bit from the point of view of facing the illness itself. (Andrologist 1)

This change was attributed to fertility specialists’ and scientists’ dedicated efforts to make oncologists aware of the existence of the fertility preservation service and their willingness to accommodate the need to avoid delaying cancer treatment.

We gave a lot of talks at different oncology hospitals, to try and get them on board. … It was also that thing for me to say, “Yes, we can do this immediately. We’ll attend the case. There’s no need for a second anaesthetic. Call me the day before, and I’ll attend”. (Scientist 1)

Active engagement with oncologists was also seen as vital in building collaborative relationships.

It’s the dialogue, and seeing patients promptly, writing good letters, ringing the oncologist, asking them what they think, keeping them informed, because it requires intense collaboration if you’re going to start chemo and you’re doing egg freezing. (Fertility specialist 1)

Through these efforts, it was thought that most oncologists in Victoria now routinely discuss the potential loss of fertility and possible options to preserve fertility with patients of reproductive age who are facing cancer treatment and refer them, where appropriate, to a clinical fertility preservation service.

It’s now, I think, almost universally accepted that all patients should have the discussion, not necessarily being referred *[for fertility preservation]* but, to us, the important thing is the conversation. Not all patients will choose an option, but it’s important that, even if they choose not to go ahead with an option, that they have really good counselling. If the oncologists can do that—or the oncology team and the oncology nurse specialist—that’s great. If they want to come to us, that’s great. (Fertility specialist 1)

### Managing urgency

To avoid delaying initiation of cancer treatment, clinics need be able to see patients at very short notice and to give them information about the available options that helps them decide whether or not to proceed with fertility preservation. Respondents, counsellors in particular, emphasised the need to be non-directive and to frame declining fertility preservation as a legitimate option.

You couldn’t make an appointment for 3 weeks’ time; it had to be dealt with that day or that week. So it was always very quick. (Counsellor 2)

I think the key concern and the key issue is usually the time limit that we have that we have to work within. Often, by the time that it’s been brought up—which is not usually the first thing that you talk about, which is understandable—the patient is already well into the planning of her chemotherapy, and so we are left with only a small amount of time. (Fertility specialist 3)

It was trying to get some sense of what they understood about what was possible with these options and what it was all about, and I think also quite strongly saying, “Yeah, you can say ‘no’ to treatment if you want to. You don’t have to do it”. And for some that was a relief. (Counsellor 2)

Informants also discussed the difficulties that can arise when young women are accompanied by their mothers. The need to ensure that the young woman is not coerced has to be balanced against the benefits of a supportive and involved parent.

One of my fears and concerns was who was actually driving the decision to store the material. (Counsellor 2)

### Managing patients’ expectations

All professions were acutely aware that careful management of patients’ expectations is an integral aspect of offering fertility preservation procedures. They perceived the need to temper hopes as an important part of their role, because of the uncertain outcomes of both cancer treatment and fertility preservation.

Informants emphasised that, for women who freeze eggs or ovarian tissue, the procedure does not only represent hope for future children but also embodies hope of survival.

If their doctor has referred them now, then that represents hope to them, and that is very powerful. They always turn up for their appointments, and they don’t make foolhardy decisions. You think, well, how can you make a decision in 2 days or 1 day? But they do, and they can, and we would never let anyone make a foolhardy decision. But it is amazing the buy-in you get from these patients, because, for them, it’s a way of looking forward. And they never whinge and complain and say, “Oh, I’ve got so much to think about; oh my god, my cancer!” They say, “Great. I want to really focus on this”. It’s almost universal, and I presume they behave the same way with their oncologist, but I don’t know. (Fertility specialist 1)

Informants underscored the importance of explaining the process of fertility preservation in a way that enabled patients, women in particular, to be fully aware of what it involves and they expressed concern that some women appear too optimistic that the procedure will guarantee future children. Counselling women and providing factual information was thus seen as a crucial part of good clinical practice.

I mean, as counsellors in the area, it was very important to us to see the people who were involved, and not just the technology, so I think we wanted to explain the technology, too, in easy-to-understand terms. And that’s what we’d done with IVF for years. … But this was different, wasn’t it, because these people weren’t infertile; they’d never been near any reproductive—they’d just been plucked out of nowhere so fast that it was crisis work. (Counsellor 1)

And we’ve always been so paranoid about making sure we don’t over-sell something and give people false hope, that we’ve possibly been overly pessimistic, I think. (Fertility specialist 1)

However, respondents did not see the need to extend counselling to men who freeze sperm before being treated for cancer.

They don’t see a counsellor; no, they don’t. Why would they? They’ve got enough counsellors from the cancer support. (Andrologist 2)

Men we didn’t see; the men we didn’t counsel for sperm freezing. That was, I think, for a number of reasons, mainly because there was no technology involved. (Counsellor 2)

### Establishing and implementing protocols, systems, and data bases

The importance of establishing protocols, systems, and data bases that allow research as well as monitoring and evaluation of clinical practice, outcomes of the procedures, and patient outcomes was emphasised by all informants. They reported from their experience that data collection in the experimental phase of fertility preservation proved inadequate once procedures were incorporated into a clinical service.

When I started, there was a database existing there, and, to me, it was just very much a telephone book: lots of patients’ names on there with a little bit of background about them, but all in a kind of ‘Notes’ field, and … you can’t do anything with text fields. You need fields that you can pull data from. (Administrator 1)

So, for research, … you need good-quality data, so that’s how I’ve sort of picked up that role. And I’m also involved with the research, and that includes the cryostorage, so I guess, over the time, we’ve developed more efficient systems. (Administrator 2)

It was viewed as essential to have comprehensive data collection that allows evidence to accumulate about the types of cancer and cancer treatments for which fertility preservation improves the chance of future conception, and about factors that influence successful use of stored reproductive material. These data would allow clinics to provide evidence-based, individualised information to patients about the options available to them.

It’s about understanding what the fertility outcomes are post-treatment. What were the treatment types that they had? How old were they? They’re the kind of fields that we felt were important to be able to draw from and analyse. … And that’s also important information for us in terms of knowing what the prognosis is for some diagnoses, and some chemotherapy strengths and whatever; for us to be able to counsel around fertility preserving options if they’re given a particular diagnosis. (Administrator 1)

The need to link clinic fertility preservation data with records in the state-based Births, Deaths and Marriages (BDM) Registry has become evident, as this provides crucial information about deaths and births after spontaneous pregnancies among people who do not return to use their stored reproductive material.

*[To avoid writing to patients who have died],* I would like to have that cross-match with the Births, Deaths and Marriages Registry. … For the ones that do have their eggs frozen, with the Births, Death and Marriages cross-match, it will be important information for us how many of those did spontaneously conceive. (Administrator 1)

Informants discussed the value of a national registry for all fertility preservation procedures and were supportive of an initiative to establish such a registry.

We’re at the point now of setting up a National Fertility Preservation Registry. The value in having a national registry, the value in getting those numbers, is so you can develop protocols and guidelines a lot quicker, is what the aim is of the national database. (Administrator 1)

### Maintaining contact with patients

Informants pointed out that storage of reproductive material can continue for decades and that it is essential to have systems for maintaining contact with patients and avoiding loss to follow-up. One commonly suggested method for ensuring up-to-date contact details was regular distribution of a newsletter to patients, which was seen as fulfilling several purposes.

This is also a way for us to keep up with our patients. There’s a little thing at the back saying, “If your information has changed, please contact us if you—”. So this is just, you know, if we can keep in contact with the patients, they can let us know if they’ve changed their address or—. (Administrator 1)

One of the purposes *[of a regular newsletter]* was to do an update of where technology was, but it was also to, I suppose, cull out people a little bit. If you’ve got return mail, you knew they’d changed their address. But also you’d get those who—you’d then find out they’d died by that method, so I think that was important. (Counsellor 1)

But part of the reason to do it *[send newsletters]* was that there was this fear that, at the time of the crisis, they actually hadn’t heard everything properly, and it was important at a later date to follow it up with more information and proper contact details and all of that stuff. (Counsellor 2)

It was also seen as important to have an active system for clinical follow-up to assess fertility and discuss potential need to use the stored material.

I think what is important is to follow up with patients, and I think we were pretty slow about that. We used to say, “Oh yeah, come back”, but now we really say “Come back for us to talk to you, and see what your ovarian function is like.” Because we really need to manage their fertility in the next 5 to 10 years after their treatment, and so we’re getting more proactive about that, so we’ll probably see more usage of eggs and tissue. (Fertility specialist 1)

## Discussion

This investigation makes an original contribution to knowledge in the opportunity it provides to learn from experts in fertility preservation. In the last 20 years, procedures for cryostorage of reproductive material to preserve potential for conception for cancer survivors have moved from being experimental to being a part of the clinical management of cancer. The insights shared by the key informants who participated in this study can be used to inform clinical practice and enhance the care and management of patients seeking fertility preservation before treatment for cancer. Learning from them is essential for what has come to be known as ‘knowledge management’ which aids the creation, transfer, and application of knowledge in an organisation [37].

To enable patients to consider fertility preservation, it is necessary for their treating oncologists to inform them about the potential impact of treatment on fertility, discuss the available options for storing reproductive material for future use, and refer them for consultation with a fertility specialist. It is known that young women and men who are diagnosed with cancer want to be informed about the consequences of gonadotoxic treatment for future fertility and about fertility preservation options but that this is not routinely offered [2, 27, 28, 38]. Studies suggest that this may in part be because health care professionals lack knowledge and resources about the available fertility preservation options and feel ill-equipped to discuss this with patients [39]. It is also possible that personal discomfort decreases the likelihood that doctors will raise fertility preservation with their patients. A study of oncologists in the UK found that lack of knowledge was one of the main barriers to discussing fertility preservation with patients [40]. Similarly, fewer than half of surveyed oncologists in the US follow the guidelines of the American Society of Clinical Oncology which recommend that all patients of childbearing age should be informed about fertility preservation [41]. Discussing fertility preservation may be particularly difficult in the context of paediatric and adolescent cancer but there is evidence that adolescents want to participate in decisions about their cancer treatment and that many are concerned about their future fertility [42]. It is suggested that educating oncologists about fertility preservation options [40, 41] as well as providing information materials about fertility preservation to patients at oncology departments and discussion prompts to physicians may improve rates of referral [43]. Informants in this study described making concerted efforts to improve oncologists’ awareness of fertility preservation options, facilitate referrals for such procedures, and accommodate the need to avoid delaying cancer treatment. These strategies were assessed as effective in increasing the number of oncologists referring people for fertility preservation procedures and improving communication and collaboration between oncologists and fertility specialists. The importance of collaborative relationships among oncology and reproductive specialists was apparent in a survey of more than 400 oncologists in Japan, where the lack of a collaborative reproductive specialist was identified as a major barrier to discussing fertility preservation with breast cancer patients [44].

Being informed about the option of fertility preservation and referred to a facility where this can be done is essential to allow people to make informed decisions about whether to proceed. Men interviewed about their experience of sperm banking before cancer treatment emphasized the vital role their oncologist had played in their decision to do so [45]. However, some who are informed and referred for fertility preservation decline the offer. Uptake of fertility preservation is influenced by individual factors such as age, parenthood status, and satisfaction with clinic care [46], perceptions of the importance of parenthood [47], cost [47], and decisional conflict [48]. Informants in our study emphasized the need for health professionals to be non-directive when discussing fertility preservation with patients and to present the option of not proceeding as a valid choice. They also cautioned that difficulties can arise when adolescents and their parents have diverging views about whether the young person facing cancer treatment should undergo fertility preservation.

After a diagnosis of cancer, confronting a decision to freeze reproductive material can be overwhelming, coinciding as it does with such an existential threat. Although counselling about the possible impact of cancer treatment on fertility would seem to be invaluable for women, men, and adolescents, it is not always provided [49, 50]. Informants in this study distinguished women’s needs from those of men, adamant that women needed counselling as part of a fertility preservation consultation but that men did not, in part because sperm freezing does not involve complex technology. However, in a study of male cancer survivors, Crawshaw [51] found that fertility-related social concerns adversely affect the well-being of men facing cancer treatment and recommended discussing these matters at the time of fertility preservation. Furthermore, Chapple et al. [28] concluded that young men who are diagnosed with cancer should be offered counselling about fertility ‘at every stage by professionals who feel comfortable talking about the subject’.

The informants’ views that optimum psychosocial care helps women to make informed decisions about whether to proceed with fertility preservation is supported by a study of more than 1000 women who had been diagnosed with cancer in their reproductive years. It found that women who had received pre-cancer treatment infertility counselling by a fertility specialist reported less long-term decision regret about having or not having preserved fertility than those who did not receive counselling [24].

Informants also viewed counselling as essential for managing potentially over-optimistic expectations. They acknowledged the need to maintain the psychological benefits of hope inherent in cryopreservation, but stressed the importance of simultaneously conveying a realistic appreciation of the chance of conception from cryopreserved material. Similar cautions have been identified in relation to pregnancy and miscarriage [49]. Nevertheless, the provision of accurate information on which to base realistic expectations, particularly for fertility preservation in women, is limited because the practice is still in its infancy and long-term outcome data are scarce [9, 52]. The limited existing evidence suggests that the rate of utilization of stored material among women who freeze oocytes or embryos before cancer treatment is low and that most post-treatment pregnancies occur spontaneously or as a result of ART with fresh oocytes [53, 54]. A recent review of 30 studies reporting reproductive outcomes for men who had stored sperm before undergoing cancer treatment found that only 8% had returned to use their stored sperm. Of those who used their stored sperm about half achieved fatherhood [55].

Because storage time for reproductive material frozen before cancer treatment can span decades, informants made it clear that accurate records are both a necessity and more than usually difficult to gather and maintain. A regular newsletter and invitations to follow-up consultations were suggested as strategies to reduce the risk of loss to follow-up and improve the ability to monitor survival rates and fertility outcomes. Birth and death registries were identified as important sources of information, contributing evidence about the safety and benefits (or lack thereof) of fertility preservation for various cancer diagnoses. However, despite strategies to maintain contact with people who store reproductive material, there are inherent challenges in long-term storage including maintaining contact and loss to follow-up. Studies of men who had stored sperm before cancer treatment report low rates of return for semen analysis to monitor fertility despite invitations to do so [56] and low disposal rates [55]. Reasons for reluctance to dispose of stored sperm include fear that cancer will recur [45], fear of being told fertility has not recovered, and being pressured to dispose of banked sperm [57].

Informants also asserted that national and international registries of large numbers of patients with diverse cancers and treatments offer the best opportunity to generate evidence about fertility after cancer treatment and the potential benefits of fertility preservation. Informants in this study are among those establishing such a registry. The Australasian Oncofertility Registry is the world’s first web-based database collecting data from oncology and fertility centres in Australia and New Zealand [www.futurefertility.com.au]. The cumulative data in this registry will allow clinicians to provide personalised advice to patients about the potential benefits of fertility preservation options which, in turn, will help patients to make informed decisions. Cancer-specific decision aids for fertility preservation may also be helpful [58].

Strengths of this study are that respondents included professionals from most disciplines involved in fertility preservation; the respondents’ extensive collective experience of fertility preservation; and that the semi-structured interviews allowed respondents to describe their own thoughts and experiences as members of a team caring for people who contemplate fertility preservation in the context of cancer. This study also has limitations: Respondents were drawn from two clinical services in one state in Australia and their views and experiences may be different from those of clinicians in other parts of Australia and in other countries; there were relatively few respondents; and no nurses participated.

## Conclusion

The collective knowledge of this study’s informants, who represent multidisciplinary teams with more than two decades of experience in fertility preservation, allows important insights into strategies that can be employed by ART clinics that offer fertility preservation in order to promote the integration of oncology and fertility care, the psychosocial care of patients who have to make difficult decisions at a very distressing time and under considerable time constraint, data recording and monitoring, and reporting of outcomes. Future research should include explorations of people’s experiences of fertility preservation to inform the development and testing of a measure of quality of fertility preservation care. Follow-up studies of the reproductive outcomes of people who store reproductive material before cancer treatment are also needed.

## Additional file

Additional file 1: Discussion Guide. (DOCX 14 kb)

## Abbreviations

ART: Assisted reproductive technology
BDM: Births, Deaths and Marriages
