# Supplementary material for: Cryopreservation of reproductive material before cancer treatment: a qualitative study of health care professionals’ views about ways to enhance clinical care
Source: BMC Health Serv Res. 2017 May 10;17:343. doi: 10.1186/s12913-017-2292-2 (PMC5424377; doi:10.1186/s12913-017-2292-2)
Supplement: Additional file 1: — Discussion Guide. (DOCX 14 kb) [file 12913_2017_2292_MOESM1_ESM.docx]

**Additional file legend**

File name: Appendix 1

Content: Discussion guide

**Discussion Guide**

Investigating development of practice for fertility preservation before cancer treatment

*Semi-Structured Interviews with past and present employees of Melbourne IVF and Monash IVF who contributed or are currently contributing to the service of fertility preservation before cancer treatment*

CONSENT:

1. Remind the participant that the aim of the research is to describe the development of practice and protocol in fertility preservation before cancer treatment.
2. Answer any questions arising from the Participant Information and Consent Form.
3. If the informant is willing to proceed, sign the consent form (for in-person interview).
4. Ask permission to record the interview.
5. If the interview is by telephone, seek verbal consent.

ROLE in relation to fertility preservation:

- Please tell me how you have contributed to providing fertility preservation to cancer patients.
  - Follow up:
    - Role? (Administrator, doctor, nurse, scientist, etc)
    - Past or current role?
    - Years?

CHANGE in fertility preservation practice:

- Please comment on how practices of fertility preservation have changed over the years.
  - Follow up:
    - The science?
    - Dealing with patients?
    - Record-keeping?
    - Development of protocols?
    - Different countries or organisations?

PATIENT USE of fertility preservation:

- What is your knowledge or impression of patient take-up of fertility preservation over the years?
- What is your knowledge or impression of patient use of their stored gametes or tissue over the years?

DOCUMENTATION:

- Can you direct us to any documentation about fertility preservation over the years?
  - Follow up:
    - Storage records that identify the reason for storage?
    - Protocols in relation to fertility preservation?

CONCLUSION:

- Is there anything else you would like to say about the development of practice in fertility preservation before cancer treatment?
  - Feel free to email or telephone me if you think of something else later.
- Would you like to have a summary of the results?
  - Email address
  - Will be some months before available
- THANK the informant for their time and very helpful contribution.
